# 1403. Baseline Electrocardiographic Profile of Patients with Severe Tetanus who developed Dysautonomia in a National Tertiary Hospital for Infectious Diseases

**DOI:** 10.1093/ofid/ofad500.1240

**Published:** 2023-11-27

**Authors:** Edna Edrada, Janice de Grace L Tulawie, Ruel O Teano

**Affiliations:** San Lazaro Hospital, Manila, National Capital Region, Philippines; San Lazaro Hospital, Manila, National Capital Region, Philippines; San Lazaro Hospital, Manila, National Capital Region, Philippines

## Abstract

**Background:**

The main causative agent of Tetanus infection is the bacterium Clostridium tetani, a gram-positive, spore-former obligate anaerobic microorganism. It continues to be an important public health concern in developing countries like the Philippines, even though it is preventable by vaccination. This infection produces a tetanospasmin which cause dysautonomia. The toxin effects on the cardiovascular system have been observed and seem to cause increasing morbidity and mortality. The paucity of literature on the degree of myocardial complications of this toxin in a local health setting geared the investigator to do this research.

**Figure:**
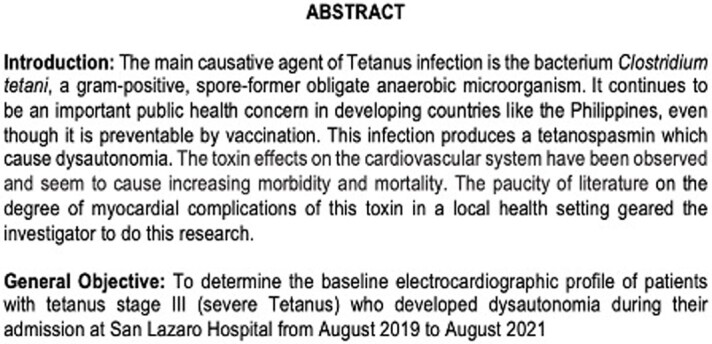

**Methods:**

This retrospective included all patients with severe tetanus infections. 325 patient’s records were retrieved. 204 met the inclusion criteria. The research investigator collected the baseline clinico-demographic of patients as to age, sex, body mass index, and presence of co-morbidities. Once collected, the investigator recorded the result of the baseline electrocardiogram of patients. Specifically, this included incidence of bradycardia, tachycardia, arrythmia and other significant electrocardiogram findings. Clinical outcome specifically length of hospital stays, and final clinical status were also determined.

**Figure:**
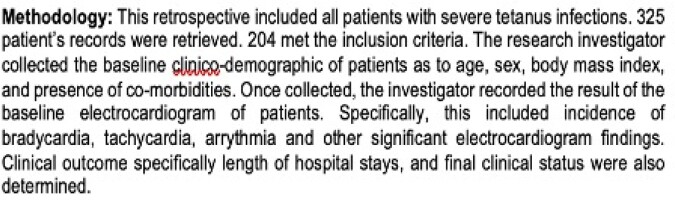

**Figure:**
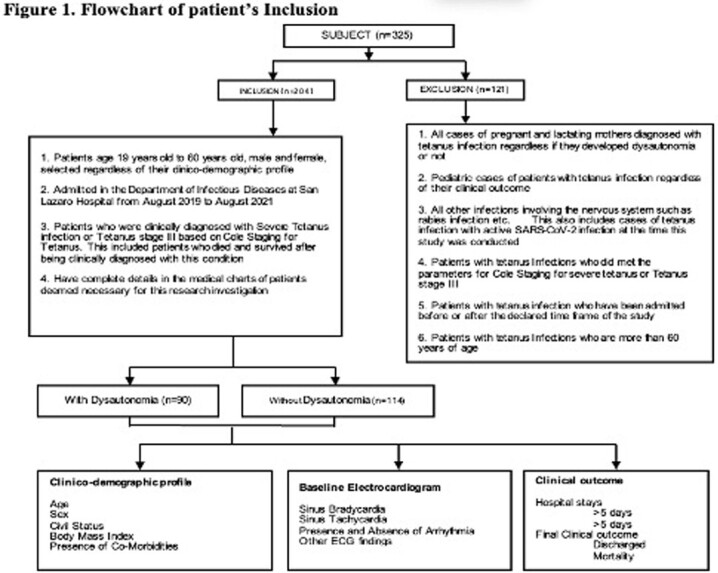

**Results:**

Of the 204 patients included, 90 (44%) developed dysautonomia, while the remaining 114 (56%) did not developed dysautonomia. Most of patients with tetanus infection were males, in the 41 to 60 years of age, married, and had normal body mass index. Comorbidities present were hypertension and diabetes mellitus. Diabetes mellitus increased the risk of dysautonomia. The most common baseline electrocardiogram finding was Sinus tachycardia (63.7%). Other arrythmia and other electrocardiogram finding such as myocardial infarction or ischemia were uncommon in this investigation.

**Figure:**
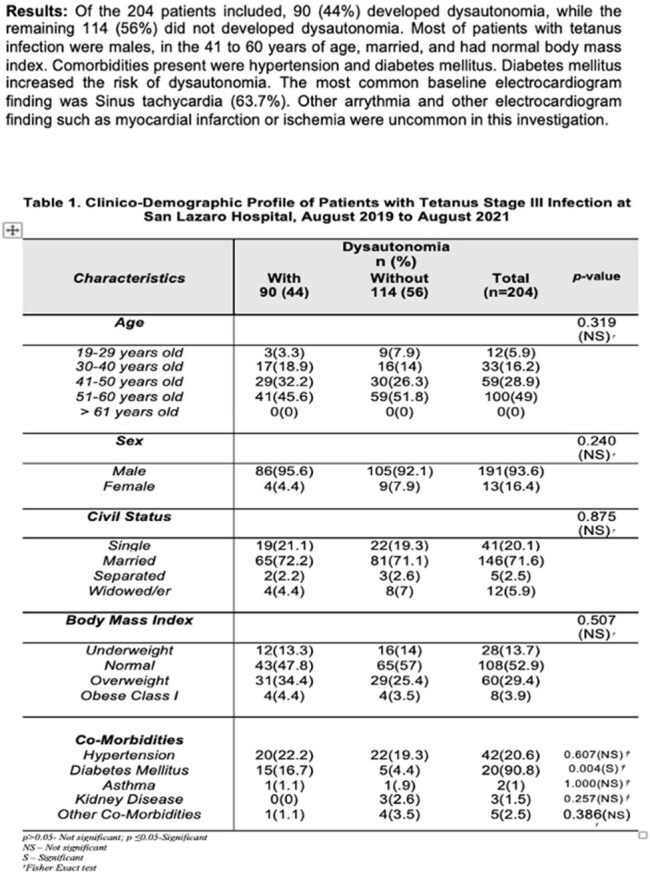

**Figure:**
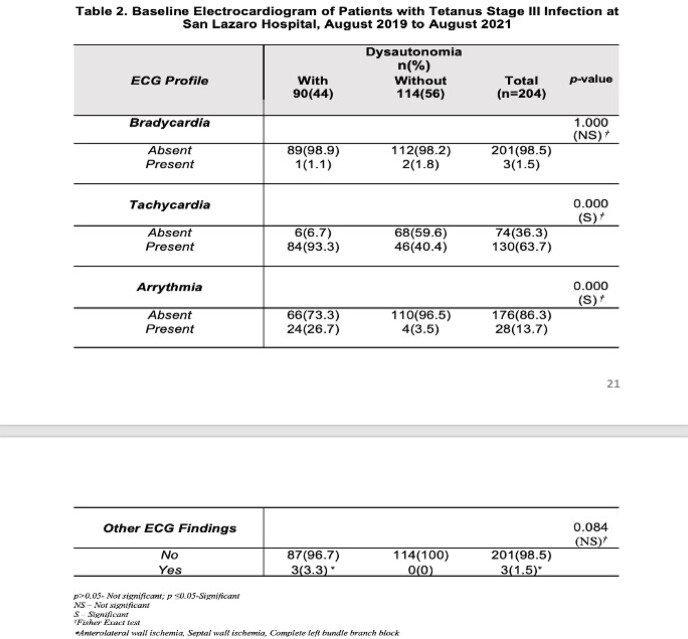

**Figure:**
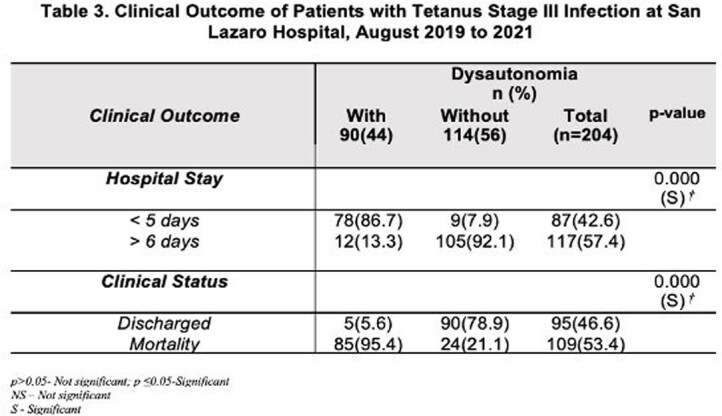

**Conclusion:**

Among the baseline electrocardiogram, finding of Sinus Tachycardia is the only result that is associated with dysautonomia and carries poor clinical outcome. As mention, diabetes mellitus increases the risk of developing dysautonomia. Patients who developed dysautonomia has shorter length of hospital stay and expired.

**Figure:**
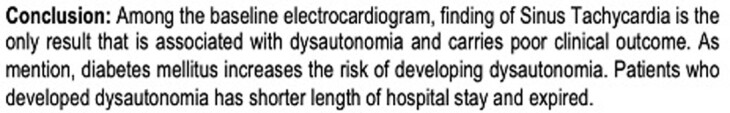

**Disclosures:**

**All Authors**: No reported disclosures

